# Molecular Detection of Shiga Toxin–Producing *Escherichia coli* O177 Isolates, Their Antibiotic Resistance, and Virulence Profiles From Broiler Chickens

**DOI:** 10.1155/ijm/9778058

**Published:** 2024-12-04

**Authors:** Tsepo Ramatla, Tshepang Motlhaping, Nkanyezenhle Ndlovu, Kealeboga Mileng, Jody Howard, George Khasapane, Taole Ramaili, Prudent Mokgokong, Jane Nkhebenyane, Rendani Ndou, Kgaugelo Lekota, Oriel Thekisoe

**Affiliations:** ^1^Unit for Environmental Sciences and Management, North-West University, Potchefstroom 2531, South Africa; ^2^Department of Life Sciences, Central University of Technology, Bloemfontein 9300, South Africa; ^3^Department of Animal Health, School of Agriculture, North-West University, Mmabatho 2735, South Africa

**Keywords:** antimicrobial resistance, *Escherichia coli* O177, shiga toxin, virulence genes

## Abstract

The World Health Organization (WHO) describes Shiga toxin–producing *Escherichia coli* (STEC) as a bacterium that can cause severe food-borne diseases. Common sources of infection include undercooked meat products and faecal contamination in vegetables. This study aimed to isolate, identify and assess the virulence and antibiotic resistance profiles of STEC isolates from broiler chicken faeces. Faecal samples were cultured, and polymerase chain reaction (PCR) was utilized to identify the isolates. Subsequently, the confirmed isolates were screened for seven virulence markers using PCR. The antibiotic susceptibility of the isolates to 13 different antibiotics was determined using the disk diffusion method. PCR was also employed to screen for antibiotic resistance genes. The *uidA* gene, which encodes the beta-glucuronidase enzyme, was detected in 62 (64.6%) of the 91 presumptively identified *E. coli* isolates. Of these, 23 isolates (37.1%) were confirmed to be *E. coli* O177 serogroup through amplification of *wzy* gene. All *E. coli* O177 isolates possessed the virulence *stx2* gene, while 65% carried the *stx1* gene. Among the *E. coli* O177 isolates, three harboured a combination of *vir + stx2 + stx1 + hlyA* genes, while one isolate contained a combination of *eaeA + stx2 + stx1 + hlyA* genes. All *E. coli* O177 isolates carried one or more antimicrobial resistance (AMR) genes, with 17 isolates (73.7%) identified as multidrug resistance (MDR). This is the first study to report the presence of *E. coli* O177 serotype from broiler chickens in South Africa. The findings reveal that broiler chicken faeces are a significant reservoir for MDR *E. coli* O177 and a potential source of AMR genes. These results underscore the importance of continuous surveillance and monitoring of the spread of AMR infectious bacteria in food-producing animals and their environments. The study also emphasizes that monitoring and control of poultry meat should be considered a major public health concern.

## 1. Introduction

Shiga toxin–producing *Escherichia coli* (STEC) is one of the most important zoonotic pathogens in the food supply chain, causing gastrointestinal complications in humans around the globe [1, 2]. It is a pathogen that causes zoonotic disease, primarily spread by cattle to humans [2]. Infection through direct contact with livestock and food consumption is possible. In general, STEC primarily occurs in ruminants and other land animals [3]. Other animal types, including chickens, are regarded as hosts, as they become infected by STEC and contribute to the spread of the disease [4].

The Shigella dysentery–producing toxin found in STEC is also referred to as Shiga-like toxin or verotoxin [5]. There are over 200 serotypes of STEC, including STEC O157, O26, O111, O103, O145, O121 and O45, which cause disease worldwide [6, 7]. The production of one or more variants of Shiga toxin (*Stx*), encoded by the *stx1* and/or *stx2* genes, distinguishes STEC from nonpathogenic *E. coli* strains isolated from the normal intestinal microbiota of healthy mammals [8]. Within Shiga toxins (*Stx*), there are two main families, *Stx1* and *Stx2*, which share 70% similarity in their amino acid sequences [6, 9, 10].

A plasmid encoding enterohaemolysin (*EhxA*) and an auto-agglutinating protein associated with *eae*-negative strains (*Saa*) are likely to produce putative accessory virulence factors in these strains [11]. Genes encoding resistance to antibiotics and heavy metals are carried on plasmids by pathogenic *E. coli*, including STEC [12, 13]. Due to its ubiquitous nature, similar niche to other enteric pathogens and ability to spread through the same route, STEC contributes to the dissemination of antimicrobial resistance (AMR) [14]. STEC exhibits antibiotic resistance through intrinsic or acquired mechanisms from other bacteria [12]. Pathogenic *E. coli* species, including STEC, carry genes encoding resistance to antibiotics and heavy metals on plasmids [12, 15]. *Escherichia coli* can spread resistance genes horizontally through plasmids to other members of the Enterobacteriaceae members [16]. When AMR genes are present on the mobile genetic elements, they increase the likelihood of AMR spreading among STEC bacteria, as well as to other bacteria, thereby reducing the available therapeutic options for humans [12].

Only one study has been conducted in South Africa to determine the prevalence of STEC in raw chicken samples, which found that 38.7% of the *E. coli* isolates were characterized as STEC [17]. However, there is no published information on the antibiotic resistance profiles exhibited by STEC in South African broiler chickens. Therefore, this study aimed to isolate and identify STEC and detect selected virulence and antibiotic resistance genes recovered from broiler chicken faeces in the North West Province of South Africa.

## 2. Materials and Methods

### 2.1. Sample Collection

Sampling was conducted as described in our previous studies [10, 18]. Briefly, a total of 480 chicken faecal samples were collected from randomly chosen broiler chickens (after evisceration) from four abattoirs (120 samples per abattoir) around Mahikeng in the North West Province. Faecal matter was picked from the caeca/rectum and placed into sterile faecal containers for laboratory processing. A total of 96 pooled faecal samples (5 chickens per pool from the same farm) were used in this study.

### 2.2. Microbiological Techniques and Analysis

Five grams of each faecal sample was mixed with 45 mL of Buffered Peptone Water (BPW) (Oxoid Ltd., Basingstoke, Hampshire, UK) enrichment broth for Enterobacteriaceae and incubated at 37°C for 24 h. The enriched broth was inoculated onto sorbitol MacConkey agar (SMA) (Merck KGaA, Darmstadt, Germany) selective plates using the spread plate method. Bacterial growth was identified after 24 h of incubation at 37°C on SMA. *Escherichia coli* appears as pink or colourless colonies on MacConkey agar because it does not ferment sorbitol or ferments it at very slow rates. The isolates were confirmed as presumptive pathogenic *E. coli* isolates after being identified phenotypically and subjected to Gram-staining as described by Ramatla et al. [10]. Pure isolates were preserved in 1.5-mL tubes containing BPW with 20% glycerol (Merck, SA) and stored at −80°C for future use.

### 2.3. Bacterial Genomic DNA (gDNA) Extraction

gDNA extraction was performed on the *E. coli* isolates cultured overnight using a ZYMO Research Quick-DNA Microbe Mini-prep Kit following the manufacturer's protocol. A NanoDrop Lite 1000 spectrophotometer (model: ThermoFisher Scientific, USA) was used to determine the concentration and purity of DNA, which was ultimately stored at −80°C until further analysis.

### 2.4. Identification of *E. coli* O177 Serogroup Using PCR Assay

DNA amplification was performed using PCR assay with primers (Table 1) targeting the 147-bp housekeeping *uidA* (*β*-D glucuronidase) gene for *E. coli*, as described by Mlangeni et al. [10]. The *E. coli* O177 serogroup-specific PCR assay was conducted to detect the *wzy* gene encoding O-antigen polymerase (Wzy) [21], using the Wzy-F and Wzy-R primers with product size of 457 bp (Table 1). The PCRs comprised 12.5 *μ*L of 2X DreamTaq Green Master Mix (New England Biolabs, USA), 8.5 *μ*L of RNase nuclease-free PCR water, 1 *μ*L of each primer (10 *μ*M each) and 2 *μ*L of template DNA. The cycling parameters consisted of an initial denaturation at 95°C for 5 min, followed by 40 cycles of 95°C for 40 sec, 60 °C for 30 sec and 72°C for 40 sec, and a final extension of 72°C for 7 min using the ProFlex PCR System (Applied Biosystems, USA). For standardization, molecular weight markers of 1 kb and 100 bp (PROMEGA, Madison, WI, USA) were used to determine the size of the PCR amplicons. A DNA-free template (nuclease-free water) was included as a negative control, while *E. coli* (ATCC:259622^TM^) was used as positive control. The results were observed under ultraviolet (UV) light using the ENDURO GDS Gel Documentation System (Labnet International Inc., US).

### 2.5. Molecular Identification of the *E. coli* O177 Virulence Genotypes by PCR Assay

PCR assays were performed to detect the presence of STEC that included *stx1* and *stx2* genes, the *eaeA* gene of enterohaemorrhagic *E. coli* (EHEC), the *lt* gene of enterotoxigenic *E. coli* (ETEC), the enteroinvasive *E. coli* (EIEC) *vir* gene, *E. coli* haemolysin (*hlyA*) and the enteroaggregative *E. coli* (EAEC) *aafII* gene (Table 1). The reaction was carried out as described above in a total volume of 25 *μ*L. The cycling parameters consisted of an initial denaturation at 96°C for 4 min, followed by 30 cycles of denaturation at 94°C for 30 sec, annealing at 55°C–64°C (Table 1) for 30 sec and extension at 72°C for 1 min using the ProFlex PCR System (Applied Biosystems, USA). A DNA-free template (nuclease-free water) was used as the negative control.

### 2.6. Antibiotic Susceptibility Pattern of the *E. coli* O177 Isolates

The antimicrobial susceptibility profiles of the isolates were determined utilizing the Kirby-Bauer disc diffusion method [23]. The following antibiotics were tested: ampicillin (AMP: 10 *μ*g), cephalothin (KF: 30 *μ*g), nalidixic acid (NA: 30 *μ*g), cefoxitin (CTX: 30 *μ*g), penicillin (P: 10 *μ*g), streptomycin (S: 10 *μ*g), ceftriaxone (CRO: 30 *μ*g), amoxicillin–clavulanic acid (AML: 20/10 *μ*g), cefotaxime (CT: 30 *μ*g), cefazolin (KZ: 30 *μ*g), tetracycline (TE: 30 *μ*g) and chloramphenicol (C: 30 *μ*g). These antibiotics were obtained from ThermoFisher, South Africa (Remel™ and Oxoid™) and were selected based on their availability and frequent use in both veterinary and human medicine in the study area [18]. The resistance patterns of the isolates to 13 different antibiotics were then interpreted following the guidelines set by the Clinical Laboratory Standards Institute [24]. The *E. coli* O177 serogroup was inoculated in nutrient broth and incubated at 37°C for 24 h. After incubation for 24 h, colonies were selected from nutrient agar, emulsified in sterile normal saline and adjusted to a 0.5 McFarland standard. The bacterial suspension was spread onto sterile Mueller–Hinton agar (MHA) plates using a sterile spreader and allowed to dry for 10 min at room temperature [10]. Antibiotic discs were placed (equally spaced) on the media, and the plates were incubated at 37 °C for 24 h. *E*. *coli* ATCC 25922 was used as a reference for quality control in antimicrobial susceptibility tests. Resistance of isolates to at least one antimicrobial agent in three or more classes was referred to as multidrug resistance (MDR) [25].

### 2.7. Detection of Antibiotic Resistance Genes

Thirteen antibiotic resistance genes (*tet*(A), *tet*(O), *tet*(W), *qnrA, qnrS, strA, strB, ermB, ampC, aadA, catI, catII* and *floR*) were screened from 23 *E. coli* O177 strains isolated from the faecal samples of broilers using PCR assays. A total of 25-*μ*L reactions were prepared, consisting of 12.5 *μ*L 2X DreamTaq Green Master Mix (New England Biolabs, USA), 2.5 mM of each primer, 2 *μ*L of template DNA and double-distilled water (ddH2O) to make the final volume. The PCR conditions were as follows: initial denaturation at 95 °C for 10 min, followed by 35 cycles of 95 °C for 30 sec, annealing at 50°C–62°C (Table 2) for 30 sec, elongation at 72 °C for 30 sec and final elongation at 72 °C for 1 min. Once amplification was completed, 8 *μ*L of the amplicon was resolved by gel electrophoresis using a 1% (w/v) agarose gel stained with ethidium bromide, and the results were observed under UV light using the ENDURO GDS Gel Documentation System (Labnet International Inc., US).

### 2.8. Data Analysis

Statistical analysis was carried out using Microsoft Excel 2016 (Microsoft Corporation, Redmond, DC, USA), and the prevalence and occurrence from this study were expressed in percentages (%). The heatmap plots of the antibiotic resistance profile were generated using ChipPlot (https:/www.chiplot.online/).

## 3. Results

### 3.1. Identification of *E. coli* O177 Isolates

A total of 91 presumptive *E. coli* isolates, based on routine bacteriological culturing methods, were recovered from 96 pooled faecal samples of broiler chickens collected from four different abattoirs. Out of 91 isolates, 62 (64.6%) were confirmed as *E. coli* using the *uidA* gene PCR assay. Furthermore, all 62 *E. coli* isolates were examined for the presence of the *wzy* gene, which is specific to the *E. coli* O177 serogroup, using a PCR assay. Out of 62 screened isolates, 23 (37.1%) were identified as belonging to the *E. coli* O177 serogroup. The majority of the *E. coli* O177 isolates (34.8%) were recovered from abattoir B, followed by abattoir A (30.4%), and lastly abattoirs C and D with 17.4% each (Table 3).

### 3.2. Detection of Virulence Genes From *E. coli* O177 by PCR Assay

**Figure 1:**
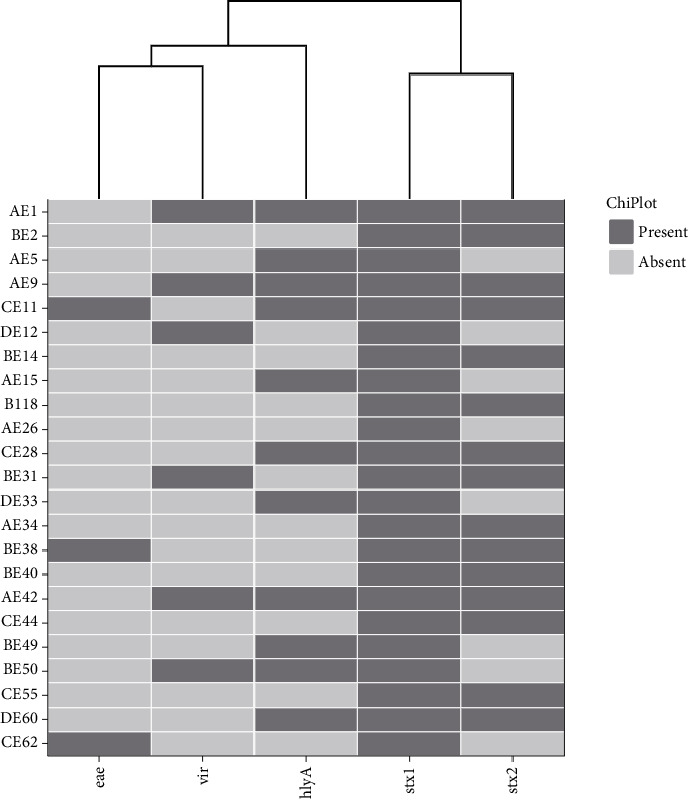
A heatmap plot showing virulence genes detected in 23 *E. coli* O177 strains isolated from broiler chickens. The black colour indicates the virulence genes that were detected in each isolate. https://www.chiplot.online/.

All five virulence factor genes (*eaeA, vir, stx2, stx1* and *hlyA*) were successfully detected from *E. coli* O177 isolates by PCR assays. Twenty-three isolates (100%) possessed the *stx1* virulence gene, followed by *stx2* (65.2%), *hlyA* (47.8%), *vir* (26%) and *eaeA* (13%) as shown in Figure 1. However, the *it* and *aafII* genes were not detected in this study. Isolates carrying more than one virulence gene were also identified, with five isolates (21.7%) containing a combination of the *stx1* and *hlyA* genes, while three isolates (13%) possessed a combination of *vir + stx2 + stx1 + hlyA* genes. Only one isolate (4.3%) contained the four-gene combination of *eaeA + stx2 + stx 1+ hlyA*.

### 3.3. AMR Profiles of the *E. coli* O177 Isolates

**Figure 2:**
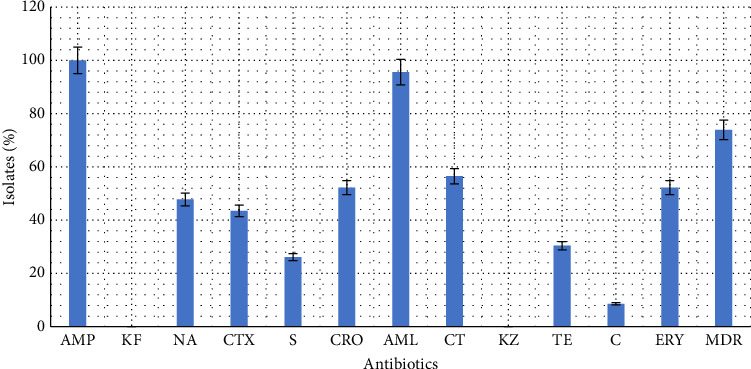
Antibiotic resistance patterns of *E. coli* O177 serogroup isolated from broiler chicken faeces. AMP: ampicillin, KF: cephalothin, NA: nalidixic acid, CTX: cefoxitin, P: penicillin, S: streptomycin, CRO: ceftriaxone, AML: amoxicillin–clavulanic acid, CT: cefotaxime, KZ: cefazolin, TE: tetracycline, C: chloramphenicol, MDR: multidrug resistant.

All the *E. coli* O177 isolates were resistant to AMP, while 22 (95.7%) isolates were resistant to AML acid (Figure 2). Conversely, all *E. coli* O177 isolates (100%) were susceptible to KZ and KF. Seventeen of the 23 isolates (73.9%) exhibited MDR phenotypes.

### 3.4. Distribution of Resistance Genes Among *E. coli* O177 Strains

**Figure 3:**
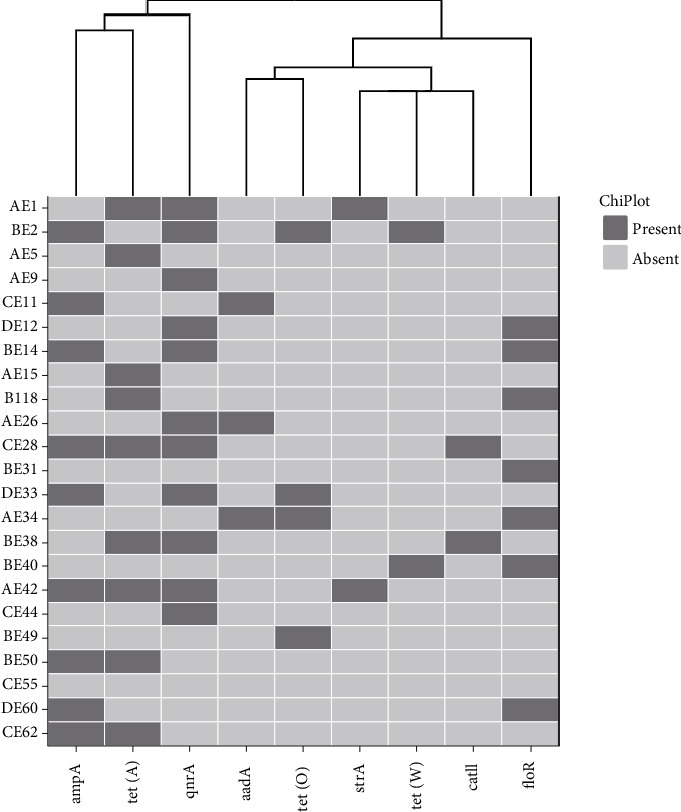
A heatmap plot showing the antimicrobial resistance genes (ARGs) detected in 23 strains of *E. coli* O177 isolated from broiler chickens. The black colour indicates the ARGs that were detected in each isolate. https://www.chiplot.online/.

All 23 *E. coli* 0177 isolates harboured resistance genes, with detection rates of *qnrA*, *tet*(A), *ampC*, *floR*, *tet*(O), *aadA*, *tet*(W), *strA* and *catII* at 47.8%, 39.1%, 39.1%, 30.4%, 17.4%, 13%, 8.7%, 8.7% and 8.7%, respectively (Figure 3). None of the isolates possessed the *qnrS, strB, ermB* or *catI* genes. A total of eight isolates (34.8%) carried two resistance genes, five isolates (21.7%) carried three resistant genes, and three isolates (13%) harboured up to four genes.

### 3.5. Coexistence of Phenotypic and Genotypic Antibiotic Resistance Among *E. coli* O177 Strains

**Figure 4:**
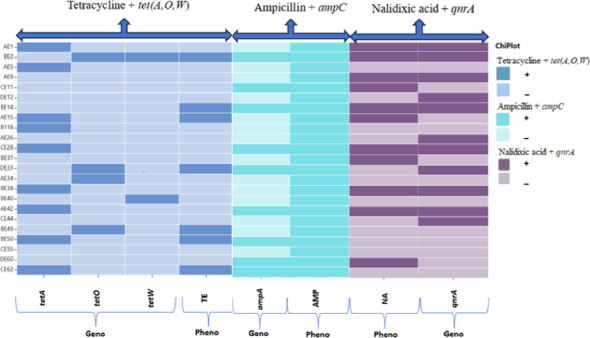
The phenotypic and genotypic antibiotic resistance profiles of *E. coli* 0177 isolates recovered from broiler chicken faecal samples. The blue colour indicates the occurrence of tetracycline resistance and the resistance genes (*tetA*, *tetO* and *tetW*); cyan indicates the presence of ampicillin and *ampC* gene and purple indicates the presence of nalidixic acid resistance and the resistance gene (*qnrA*). Geno = genotypic and Pheno = phenotypic. https://www.chiplot.online/.

Seven *E. coli* O177 isolates (30.4%) that exhibited phenotypic resistance to NA carried the *qnrA* gene. Three isolates resistant to TE, harboured *tet*(A), while another three carried the *tet*(O), and one isolate possessed both *tet*(O) and *tet*(W) genes. Nine isolates resistance to AMP carried the *ampC* gene (Figure 4).

## 4. Discussion

The investigation through the amplification of the *wzy* gene revealed that 28.3% of the isolates were *E. coli* O177. The proportion is lower than the 41.09% found in a study by Montso, Mlambo and Ateba [21], which focused on cattle in South Africa's North West Province. On the contrary, the prevalence of *E. coli* O177 in broiler chickens in this study was 37.1%, which is higher than a previous report where this serogroup was detected in broiler chickens from Poland [31]. From a public health perspective, this infection rate is significant, even though the overall prevalence was not substantial.

To verify the production potential of Shiga-like toxins (STEC) in this study, the *stx2* and *stx1* genes were detected in 100% and 65.2% of the *E. coli* O177 isolates, respectively. These toxins are associated with EHEC activity. The results of this study are similar to a previous investigation by Karama et al. [7], which was conducted on 140 isolates recovered from beef cattle in Gauteng Province, South Africa. In that study, the prevalence of *stx2* (34.3%) gene was higher than the 4.3% prevalence of the *stx1* gene. Another study conducted in Gauteng Province by Onyeka et al. [32] revealed that *stx1* had the highest prevalence (24%), followed by *stx2* (17%). Our findings are consistent with Montso, Mlambo and Ateba [21], who reported 11.2% prevalence of the *stx2* gene from 376 *E. coli* O177 isolates recovered from dairy and beef cattle faeces in the North West Province, South Africa. Additionally, a study by Zarei et al. [17] on chicken samples in South Africa reported *stx1* and *stx2* prevalence rates of 7.47% and 33.3%, respectively, among the 93 *E. coli* O157 isolates. In contrast, Karama et al. [7] observed a higher prevalence of the *stx1* gene (84%) than *stx2* (29%) in human samples collected in South Africa. This variation in gene frequency could be due to differences in sample sizes. It is well established that Shiga toxins are critical to the virulence of STEC in humans and can cause severe gastroenteritis [33]. These studies highlight the importance of the *stx* genes as key virulence factors of *E. coli* strains in South Africa. However, this is the first study to report the presence of Shiga-like toxins in *E. coli* O177 strains isolated from broiler chickens. Consuming raw or undercooked meat contaminated with STEC strains has been linked to multiple outbreaks of bacterial food-borne illness [17].

This study also detected the presence of *eaeA, vir* and *hlyA* virulence genes in the *E. coli* O177 isolates. The *eaeA* gene encodes intimin, a protein involved in the formation of attaching and effacement lesions. The association of STEC strains from this study carrying *eaeA* indicates their potential to cause severe diseases in human [33, 34]. The presence of the *eaeA* gene suggests that these strains may form intimate attachments to epithelial host cells, resulting in “attaching and effacing” lesions. Among the 23 *E. coli* O177 strains, only 13% possessed the *eaeA* gene. Montso, Mlambo and Ateba [21] and Onyeka et al. [32] reported the prevalence of the *eaeA* gene in *E. coli* O177 isolates from cattle at 7.25% and 70%, respectively. Additionally, 47.8% of *E. coli* O177 isolates harboured the *hlyA* (*α*-haemolysin) gene. In comparison, Hashemizadeh et al. [35] reported a lower prevalence of *hlyA* gene (28.8%) in *E. coli* isolates from patients with urinary tract infections in Iran.

This study recorded three isolates that possessed a combination of *vir+ stx2+stx1+hlyA* and one isolate that harboured a combination of four virulence genes (*eaeA + stx2 + stx1 + hlyA*). The detection of Shiga toxin among *E. coli* O177 isolates from this study may offer insight into the potential health risk and these isolates pose to humans. The virulence gene combinations observed in this study are similar to those reported by Jajarmi et al. [36] from *E. coli* isolated from cattle faeces in Iran and observed by Montso et al. [21] in *E. coli* O177 isolates from cattle faeces in South Africa. To generate comparable data on the prevalence and trends of STEC in chicken feed, chicken and the environment, further research is needed.

One of the most concerning issues of this century is the emergence of antibiotic-resistant microorganisms [25, 37], and hence, the phenomenon is a current public health issue that is widely discussed [38]. In this study, we examined the frequency of antibiotic resistance traits in *E. coli* O177 isolates from broiler chicken faeces. This study revealed that all the *E. coli* O177 isolates were resistant to AMP, while 95.7% were resistant to AML acid, 56.5% to CT, 52.2% to CRO and 52.2% to erythromycin. These results are in contrast with a previous study on *E. coli* O177 isolates from cattle, which reported higher resistance rates to erythromycin (63.84%), AMP (21.54%), TE (13.37%), S (17.01%) and kanamycin (2.42%) in South Africa [21]. Furthermore, other studies such as those conducted by Ranjbar et al. [39] in Iran recorded resistance to AMP (100%), gentamicin (100%) and TE (96.87%) in raw milk and traditional dairy products. Similarly, Abebe et al. [40] observed that 100% of the *E. coli* O157: H7 isolates from foods of bovine origin were resistant to P g, vancomycin and oxacillin, which contradicts observations by Thungrat et al. [41], who reported resistance to KF (98%) and KZ (11%). Geographical location and sample type may explain these discrepancies. Lower resistance (30.4%) to TE was observed in broiler chickens compared to a previous study conducted in Ethiopia, where 96.0% of *E. coli* O157: H7 isolates from foods of bovine origin were resistant to TE [40]. These findings demonstrate that multiple AMR genes have accumulated in domestic animal *E. coli* isolates, raising concerns over the spread of extensively drug-resistant organisms.

Phenotypically, 13.1% of *E. coli* O177 isolates were resistant to erythromycin, but interestingly, this study did not detect the *ermB* gene, which encodes for erythromycin resistance. In contrast, other studies have shown that the *ermB* gene is the most prevalent (38%) in *E. coli* isolates from urban waterways in the USA [42]. Among Gram-negative bacteria, *tetA, tetB, tetC, tetD* and *tetG* genes encode the energy-dependent efflux pump system, with *tetB* and *tetA* genes commonly detected in *E. coli* strains [43]. In this study, the prevalence of TE resistance genes in *E. coli* O177 isolates was ranked as follows: *tet*(A) (39.1%), *tet*(O) (17.4.1%) and *tet*(W) (8.7%), respectively. Five isolates (21.7%) showed phenotypic resistance to TE and possessed the *tet* gene, in contrast to previous studies in cattle, where all isolates exhibited phenotypic resistance to TE, but the *tet*(W) gene was not detected [21, 43].

## 5. Conclusion

This is the first study to report the prevalence of STEC O177 isolates from broiler chicken faeces in South Africa. The presence of the *stx2 + eaeA* virulence gene combination in 13.04% of the STEC O177 isolates indicates the potential health risk of transmission to humans. The current study confirms that healthy chickens serve as reservoirs for antibiotic-resistant *E. coli* with virulence factors capable of causing infection. The presence of MDR in nearly all STEC O177 isolates highlights the potential therapeutic challenges if humans become infected by these isolates. Continued surveillance and research are required in South Africa to track the emergence and spread of multiresistant STEC O177 across different provinces. Additionally, a “One Health” approach should be developed by the agricultural and human health sectors to manage the spread of this STEC O177 strain in the country.

## Figures and Tables

**Table 1:** PCR assay primer sequences used for the confirmation of *E*. *coli* isolates and detection of their virulence genes.

Target gene	Primer	Sequence of primers (5–3′)	Amplification length (bp)	Annealing temp. (°C)	Reference
*Virulence genes*					
*lt*	FW	GCACACGGAGCTCCTCAGTCTCC	218	56	[19]
	RV	TTCATCCTTTCAATGGCTTT			
*aafII*	FW	CACAGGCAACTGAAATAAGTCTGG	378	56	[19]
	RV	ATTCCCATGATGTCAAGCACTTC			
*vir*	FW	AGCTCAGGCAATGAAACTTTGAC	822	61	[19]
	RV	TGGGCTTGATATTCCGATAAGTC			
*eaeA*	EAE1	TCAATGCAGTTCCGTTATCAGTT	450	55	[19]
	EAE2	GTAAAGTCCGTTACCCCAACCTG			
*stx1*	EVT1	CAACACTGGATGATCTCAG	349	55	[20]
	EVT2	CCCCCTCAACTGCTAATA			
*stx2*	EVS1	ATCAGTCGTCACTCACTGGT	110	55	[20]
	EVS2	CTGCTGTCACAGTGACAAA			
*hlyA*	HlyA-F	GCATCATCAAGCGTACGTTCC	534	64	[21]
	HlyA-R	AATGAGCCAAGCTGGTTAAGCT			

*Identification*					
*uidA*	UidA-F UidA-R	AAAACGGCAAGAAAAAGC ACGCGTGGTTACAGTCTTGCG	147	60	[22]
*wzy*	Wzy-F Wzy-R	GGTCAGGAGCATGGAGCATT AATCCATCCGGTGTATCGGC	457	55	[21]

**Table 2:** List of antibiotic resistance gene primers and conditions used in this study.

Class	Target gene	Primer	Primer sequence (5′ ⟶ 3′)	Amplicon size (bp)	Annealing temp (°C)	References
Tetracycline	*tet*(A)	TETA-F TETA-R	GCGCTNTATGCGTTGATGCA ACAGCCCGTCAGGAAATT	387	62	[26]
	*tet*(O)	TETO-F TETO-R	ACGGARAGTTTATTGTATACC TGGCGTATCTATAATGTTGAC	171	60	[26]
	*tet*(W)	TETW-F TETW-R	GAGAGCCTGCTATATGCCAGC GGGCGTATCCACAATGTTAAC	168	50	[26]

Erythromycin	*erm*B	ERMB -F ERMB -R	GCATTTAACGACGAAACTGGCT GACAATACTTGCTCATAAGTAATGGT	573	61	[26]

Chloramphenicol						[26]
	*cat*I	catI-F catI-R	GGTGATATGGGATAGTGTT CCATCACATACTGCATGATG	349	60	[26]
	*cat*II	catII-F catII-R	GATTGACCTGAATACCTGGAA CCATCACATACTGCATGATG	567	60	[26]
	*flo*R	FloR-F FloR-R	CGCCGTCATTCCTCACCTTC GATCACGGGCCACGCTGTGTC	215	50	[27]

*β*-lactam	*amp*C	AmpC -F AmpC R	GTGACCAGATACTGGCCACA TTACTGTAGCGCCTCGAGGA	822	61	[28]

Quinolone	*qnr*A	qnrA-F qnrA-R	ATTTCTCACGCCAGGATTTG GAGATTGGCATTGCTCCAGT	413	56	[29]
	*qnr*D	qnrD-F qnrD-R	GCTGGAGCTTGTCAGGGATT TGCTGCGAGATATCATGCGT	585	59	[29]
	*qnrS*	qnrS-F qnrS-R	CCCCATGCCCGAAGTTATCA ACTGCTTGGAGTGTGTTGGT	457	53	[30]

**Table 3:** The number of samples collected per abattoir and the total of pooled samples.

Abattoirs ID	Samples collected	Pooled samples	*E. coli* isolates (%)	*E. coli* O177 (%)
A	120	24	19 (30.6%)	7 (30.4%)
B	120	24	13 (20.9%)	8 (34.8%)
C	120	24	14 (22.5%)	4 (17.4%)
D	120	24	16 (25.8%)	4 (17.4%)
Total	480	96	62	23

## Data Availability

The data used to support the findings of this study are included in the article.

## Nomenclature

AMP: Ampicillin
KF: Cephalothin
NA: Nalidixic acid
CTX: Cefoxitin
P: Penicillin
S: Streptomycin
CRO: Ceftriaxone
AML: Amoxicillin–clavulanic acid
CT: Cefotaxime
VA: Vancomycin
KZ: Cefazolin
TE: Tetracycline
C: Chloramphenicol
*E. coli*: *E. coli*
STEC: Shiga toxin–producing *E. coli*
EHEC: Enterohemorrhagic *E. coli*
ETEC: Enterotoxigenic *E. coli*
EIEC: Enteroinvasive *E. coli*
EAEC: Enteroaggregative *E. coli*
AMR: Antimicrobial resistance
MDR: Multidrug resistance
CLSI: Clinical and laboratory standards institute
WHO: World Health Organization
DNA: Deoxyribonucleic acid
SMA: Sorbitol MacConkey agar
PCR: Polymerase chain reaction
